# Assessing attitudes towards insulin pump therapy in adults with type 1 diabetes: Italian validation of the Insulin Pump Attitudes Questionnaire (IT-IPA questionnaire)

**DOI:** 10.1007/s00592-023-02046-7

**Published:** 2023-02-20

**Authors:** Rossella Messina, Liliana Indelicato, Marica Iommi, Maddalena Trombetta, Timm Roos, Norbert Hermanns, Annamaria Di Sipio, Maria Pia Fantini, Vincenzo Calvo

**Affiliations:** 1 Department of Biomedical and Neuromotor Sciences, University of Bologna, Bologna, Italy; 2 Division of Endocrinology, Diabetes and Metabolic Diseases, AOUI Verona, Italy; 3 Division of Endocrinology, Diabetes and Metabolic Diseases, Department of Medicine, University of Verona, Verona, Italy; 4 Research Institute of the Diabetes Academy Mergentheim (FIDAM), Bad Mergentheim, Germany; 5 Department of Philosophy, Sociology, Pedagogy, and Applied Psychology, University of Padova, Padova, Italy

**Keywords:** Insulin pump therapy, Type 1 diabetes, Psychometric validation, Psychosocial aspects, Attitudes

## Abstract

**Aims:**

The aim of the study was to adapt the German version of the insulin pump therapy (IPA) questionnaire to Italian (IT-IPA) and to evaluate its psychometric properties in adults with type 1 diabetes.

**Methods:**

We conducted a cross-sectional study, data were collected through an online survey. In addition to IT-IPA, questionnaires evaluating depression, anxiety, diabetes distress, self-efficacy, and treatment satisfaction were administered.

The six factors identified in the IPA German version were assessed using confirmatory factor analysis; psychometric testing included construct validity and internal consistency.

**Results:**

The online survey was compiled by 182 individuals with type 1 diabetes: 45.6% continuous subcutaneous insulin infusion (CSII) users and 54.4% multiple daily insulin injection users.

The six-factor model had a very good fit in our sample. The internal consistency was acceptable (Cronbach’s α = 0.75; 95% IC [0.65–0.81]).

Diabetes treatment satisfaction was positively correlated with **a** positive attitude towards CSII therapy (Spearman’s rho = 0.31; *p* < 0.01), less Technology Dependency, higher Ease of Use, and less Impaired Body Image. Furthermore, less Technology Dependency was associated with lower diabetes distress and depressive symptoms.

**Conclusions:**

The IT-IPA is a valid and reliable questionnaire evaluating attitudes towards insulin pump therapy. The questionnaire can be used for clinical practice during consultations for shared decision-making to CSII therapy.

## Introduction

The use of technological devices in management of type 1 diabetes is widely increased in the past few years and has been shown to improve Glycaemic Control, health-related quality of life, and treatment satisfaction [1]. Furthermore, insulin pump users reported benefits such as more Flexibility, freedom [2], and decreased stress [3]. Despite these several benefits, insulin pump therapy is still limited [4, 5]. In Italy, a survey in 2020 showed that only 18,1% of adults with type 1 diabetes were using an insulin pump [6]. In a qualitative study, Ritholtz and colleagues identified some themes associated with reluctance to transition to insulin pump therapy [7]. These included the potential impact on diabetes self-care, emotional reactions to the insulin pump, body image, and social acceptance. However, the effect of the transition from multiple daily insulin injections (MDI) to insulin pumps on perception and treatment satisfaction has been less studied [8].

Moreover, a large body of research over the past decade has shown several clinical benefits in people on insulin pump therapy. However, such benefits depend on the successful uptake and continued use of these devices [9]. Thus, it is important to consider the complexity of diabetes management. Both physical and psychosocial challenges significantly impact and affect the success of any diabetes device and must be adequately addressed [8].

Wearing an insulin pump requires adjusting insulin dose according to meals, physical activity, and responding to fluctuations in glucose levels [9]. For these reasons, patient motivation and willingness to undergo insulin pump therapy are needed to achieve optimal results from new technologies. The assessment of psychological factors involved in adherence to an insulin pump is very important to identify patients’ needs, desires, and skill levels because there is no “one-size-fits-all” approach to technology, as reported by Standards in Medical care in Diabetes [10].

Psychological characteristics and attitudes of individuals affected by type 1 diabetes may play a significant role in influencing adherence and efficacy of Continuous Subcutaneous Insulin Infusion (CSII) therapy. Some of the barriers identified in the literature are related to the potential negative impact on body image and the feeling of technology dependence [11, 12]. Moreover, according to the technology acceptance model [13], people accept to use a technological device if this is perceived as useful and easy to use. For this reason, it might be important to evaluate expectations and experiences related to CSII therapy. Addressing psychological aspects in clinical practice and identifying possible unfavourable attitudes in diabetes care is useful to evaluate attitudes towards insulin pump therapy to promote shared decision-making between the person with diabetes and the consultant and to avoid dropout of the device during the time.

In Italy, a questionnaire evaluating attitudes towards diabetes technological device utilization does not yet exist. The Insulin Pump Attitudes Questionnaire (IPA) [14] has been developed in 2019 in Germany to assess expectations and experiences towards insulin pump therapy. The IPA can differentiate between pump users and non-pump users, is a valid and reliable new instrument to assess attitudes towards CSII therapy, and provides a comprehensive analysis of benefits, barriers, and technical problems of CSII therapy. The IPA questionnaire may be used in clinical practice to address the different attitudes of pump users and also in people that are considering using it. For this reason, this study aimed to validate the Italian version of the IPA questionnaire in a sample of adults with type 1 diabetes, to analyse its psychometric properties, and to explore correlations with psychological aspects.

## Methods

### Study design and participants

In this cross-sectional study, data were collected through an online survey conducted from February to August 2021. Participants were enrolled using a chain sampling method. They were invited to take part in a study on the use of technology in the management of diabetes. In addition to the Italian version of the IPA questionnaire, the following questionnaires were also administered to the participants: Patient Health Questionnaire (PHQ-9) to evaluate depression, Generalized Anxiety Disorder (GAD-7) questionnaire to assess anxiety, Problem Areas in Diabetes Scale (PAID-5) for diabetes distress, the Italian version of Diabetes Management Self-Efficacy Scale (DMSES) to estimate self-efficacy, and Diabetes Treatment Satisfaction Questionnaire (DTSQ) for treatment satisfaction. Sociodemographic characteristics (sex, age, living with, level of education, occupational status), type of device used (MDI or CSII), years of actual devices utilization, duration of the disease (years since the onset of type 1 diabetes), last detection of HbA1c (mmol/mol), body mass index (BMI), and self-reported clinical information (events of hypoglycaemia in the last year, events of ketoacidosis in the last year, and presence of diabetes-related complications) were also self-reported in a preliminary interview.

Inclusion criteria were: age ≥ 18 years; diagnosis of type 1 diabetes more than 6 months.

Exclusion criteria were: type 2 diabetes.

The study was approved by the Ethical Committee for Psychological Research at the University of Padova.

### Instruments

#### The Italian version of the Insulin Pump Attitudes Questionnaire, IT-IPA

The questionnaire is composed of 26 items. The German version identified, through exploratory and confirmatory factor analysis, six subscales: Glycaemic Control (GC) Flexibility (Fle), Impaired Body Image (IBI), Technology Dependency (TD), Ease of Use (EoU), and Functionality (Fun). The IPA total score correlates significantly with diabetes distress, self-efficacy, diabetes empowerment, psychological well-being, and treatment dissatisfaction, supporting criterion validity. The total score ranges from 0 to 104, with higher scores indicating a more positive attitude towards CSII therapy [14]. For this study, an Italian version of the IPA questionnaire has been developed.

#### Patient Health Questionnaire*, PHQ-9*

The PHQ-9 questionnaire, comprising 9 items, is used to investigate the presence of depressive symptoms for a possible diagnosis of major depression. Scores range from 0 to 27, with cut points 5, 10, 15, and 20, indicating, respectively, mild, moderate, moderately severe, and severe levels of depressive symptoms [15, 16].

The PHQ-9 has proven adequate psychometric characteristics, reliability, and validity in people with diabetes [17].

#### Generalized Anxiety Disorder*, GAD-7*

This questionnaire is composed of 7 items to measure levels of anxiety in the last 15 days. The score ranges from 0 to 21, with cut points 5, 10, and 15 indicating, respectively, mild, moderate, and severe levels of anxiety [18].

The GAD-7 has shown convincing psychometric properties, reliability, and validity, in the general population [19] and different samples of patients [20], and it has been used to screen for generalized anxiety disorder in young adults with diabetes [21].

#### Problem Areas in Diabetes Scale*, PAID-5*

This scale is used to investigate diabetes distress and the emotional burden related to diabetes management [22, 23]. The PAID-5 has been validated in Italian in the BENCH study [24]. It includes 5 items, with a total score ranging from 0 to 20. A score ≥ 8 indicates the presence of diabetes distress [22].

The PAID-5 has resulted to be a psychometrically adequate, reliable, and valid measure of diabetes-related emotional distress [22, 24].

#### The Italian version of the Diabetes Management Self-Efficacy Scale, IT-DMSES

The Italian version of the DMSES measures self-efficacy in diabetes management [25]. IT-DMSES has good psychometric characteristics and consists of 15 items divided into two factors: factor 1, self-efficacy in diabetes management, related to self-perception of patient’s ability to manage the activities related to diabetes management (e.g. medication adherence, Glycaemic Control), and factor 2, related to self-efficacy in lifestyle management (e.g. eating behaviours, physical activities). Scores per factor range from 0 to 10, indicating low (0–3), mid (4–6), and high (7–10) levels of self-efficacy.

#### Diabetes Treatment Satisfaction Questionnaire, DTSQ

The DTSQ is a valid and reliable questionnaire of 8 items measuring patients’ diabetes treatment satisfaction [26, 27]. Its scores range from 0 to 36. There is a total score and two scales for the evaluation of perceived hypo and hyperglycaemia episodes. For this study, we used the total score.

### Face and content validity

The IPA questionnaire was translated to Italian and then back-translated to German by a bilingual German native speaker [28, 29]. To improve the intelligibility of the questionnaire, items were reviewed by a panel of experts including 2 diabetologists and then revised by the research team. No major revisions were done by the 2 experts.

The version agreed with the team was then administered (RM, LI) to a pilot sample of 5 people with type 1 diabetes in CSII therapy, aged between 18 and 25. A cognitive interviewing methodology was applied to assess the perception, usefulness, and interpretation of each item [30, 31]. During completion, people were asked to provide comments on items and terminology employed, and comments were reported in field notes. Results of the supervised pilot administration of the instrument indicated that patients had some comments concerning items 2, 3, 12, 16, 19, 22, and 24, and in particular in item 2, the adjective “spontaneous” has been suggested by the pilot sample to be replaced with “natural” (see Table 1). The time of administration ranged from 5 to 10 min. Since the scale aims to measure expectations and/or experiences related to insulin pump utilization, people who did not use insulin pumps were asked to answer by imagining how it might be. For example, if they never used the pump, they may know the insulin pump advantages in the absence of experience. The pilot group reported that the questionnaire was interesting and introduced all the contents related to CSII therapy; they stated also that the questionnaire would be most appropriate for people who during the course of diabetes had experiences with diabetes technological devices.

**Table 1:** Comments to the items during pilot administration

Items	Comments
2	The adjective “spontaneous” has been replaced with “natural” for misleading interpretation
3	The substantive “preparation” for the sport has been commented: what does preparation for the sport mean? Less modifications of the insulin dosage? it might include modifications on diet and in particular introducing snacks before sport activities
12	This is a sentence that is comprehensive only for people that are confident on using the CSII or have received yet some technical information
16	The word “ill” is a bad term on using with patients. Interpretations evocated by this term are: (i) illness makes me fill conscious of the gravity of this conditions and it scares me; (ii) It makes me fill more the burden related to diabetes management
19	The pump alarms make me feel worried means also that can disturb my sleep (same of item 22)
22	The pump alarms may disturb partner’s sleep and this would be annoying for the relationship
24	The adjective “mine” does not take into account people that are in MDII therapy

### Statistical analysis

Demographic and clinical characteristics were summarized with absolute frequencies and percentages for categorical variables, with mean and standard deviation (SD) or median and interquartile range (IQR) for continuous variables, according to the shape of data distribution.

MDI and CSII users were compared using the t-test for continuous variables with a normal distribution, Mann–Whitney test for asymmetric continuous variables, and χ^2^-test for categorical variables.

Confirmatory factor analysis (CFA) was performed to compare the goodness of fit of the six-factor solution identified by Bergis et al. [14] in a study with 452 people with type 1 diabetes. In this analysis, items were used as categorical variables, and factors were estimated using a robust weighted least-squares estimator. The models’ goodness of fit was evaluated considering the Tucker–Lewis Index (TLI), the Comparative Fit Index (CFI), the root mean square error of approximation (RMSEA), and the standardized root mean square residual (SRMR). TLI and CFI values > 0.90 reflect an acceptable fit and values > 0.95 imply a very good fit. For both RMSEA and SRMR values < 0.05 indicate close model fit; values up to 0.08 suggest a reasonable error of approximation in the population, and values > 0.10 indicate poor fit. The fit indices were assessed collectively, so that a single index that fell just outside the acceptable range was not necessarily considered to reflect poor model fit, provided that the other statistics indicated good model fit.

Internal consistency was assessed using Cronbach’s α coefficient and its relative 95% confidence interval (95% CI). The internal consistency was considered excellent when α coefficient ≥ 0.9, good when 0.8 ≤ α < 0.9, acceptable when 0.7 ≤ α < 0.8, questionable when 0.6 ≤ α < 0.7, poor when 0.5 ≤ α < 0.6, and unacceptable when α < 0.5 [32].

Spearman rank correlations were computed to examine convergent (DTSQ, DMSES) and discriminant validity (PHQ-9, GAD-7, PAID-5) based on data from the first assessment time point.

All analyses were performed using R version 4.1.0, and the significance level was set at *p* < 0.05.

## Results

### Study sample

The online survey was compiled by 182 individuals with type 1 diabetes: 99 (54.4%) MDI users and and 83 (45.6%) CSII users. About 53.3% of the total sample were females and the median age was between 27 and 29 for MDI and CSII users (Table 2).

**Table 2:** Demographic and clinical characteristics of the study sample (*n* = 182)

Sample characteristics	Total (*n* = 182)		MDI (*n* = 99)		CSII (*n* = 83)		Test	p
	n	%	n	%	n	%		
Sex (female)	97	53.3%	49	59.0%	48	48.5%	2.02^#^	0.155
Age, median [IQR]	29	[23–39]	27	[23–40]	29	[23–39]	4351^*^	0.493
Early adults	79	43.4%	44	44.4%	35	42.2%	0.10^#^	0.758
Living with							1.78^#^	0.617
Alone	16	8.8%	11	11.1%	5	6.0%		
Parents	59	32.4%	33	33.3%	26	31.3%		
Partner/spouse	89	48.9%	46	46.5%	43	51.8%		
Others	18	9.9%	9	9.1%	9	10.8%		
Level of education							1.94^#^	0.584
Middle school	4	2.2%	3	3.0%	1	1.2%		
High school	103	56.6%	57	57.6%	46	55.4%		
College	61	33.5%	30	30.3%	31	37.3%		
Post-degree	14	7.7%	9	9.1%	5	6.0%		
Occupational status							4.91^#^	0.427
Student	37	20.3%	18	18.2%%	19	22.9%		
Working student	8	4.4%	2	2.0%	6	7.2%		
Unemployed	13	7.1%	8	8.1%	5	6.0%		
Part-time job	19	10.4%	9	9.1%	10	12.0%		
Full-time job	102	56.0%	60	60.6%	42	50.6%		
Retired	3	1.6%	2	2.0%	1	1.2%		
Time of MDI/CSII utilization (years), median [IQR]	3	[1–5]	3	[1–4]	3	[2–6]	4917^*^	**0.006**
Duration of the disease (years), median [IQR]	16	[9–24]	14	[8–24]	17	[11–24]	4587^*^	0.176
HbA1c (mmol/mol), median [IQR]	60	[52–75]	65	[54–78]	55	[49–70]	3106^*^	**0.005**
BMI, median [IQR]	22.5	[21.6–24.6]	22	[20.7–24.6]	23	[21.4–24.8]	4515^*^	0.251
Events of hypoglicaemia in the last year	25	13.7%	15	15.2%	10	12.0%	0.37^#^	0.545
Events of ketoacidosis in the last year	15	8.2%	8	8.1%	7	8.4%	0.01^#^	0.931
Presence of diabetes-related complications	35	19.2%	21	21.2%	14	16.9%	0.55^#^	0.459

The significant p values are shown in bold

*MDI* multiple daily insulin injections, *CSII* Continuous subcutaneous insulin infusion, *IQR* interquartile range, *mmol/mol* millimoles per mole, *BMI* body mass index

* Mann–Whitney U test; # Chi-square test; § t-test

The two groups differed for years of actual devices utilization and HbA1c: CSII had more years of device utilization (*p* = 0.006) and lower level of HbA1c (*p* = 0.005), while no differences were observed for the presence of hypoglycaemia or ketoacidosis episodes in the last year nor the presence of diabetes-related complications.

**Fig. 1:**
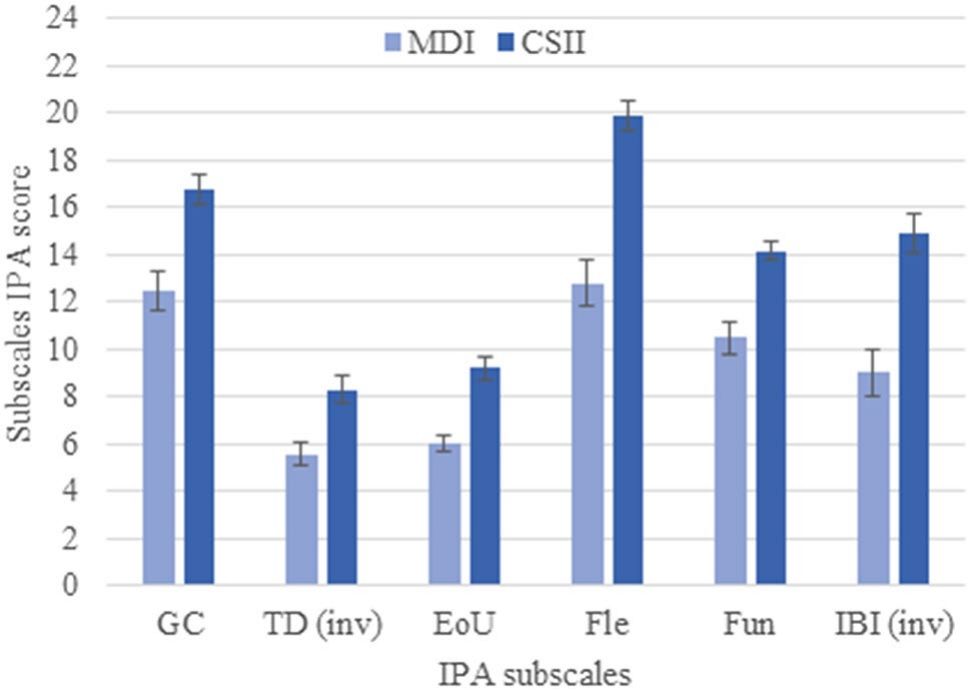
Comparison of IPA subscale scores between CSII users and MDI users. *IPA* Insulin Pump Attitudes, *IPA* subscales *GC* Glycaemic Control, *TD (inv)* Technology Dependency (inverted score), *EoU* Ease of Use, *Fle* Flexibility, *Fun* Functionality, *BI (inv)* Impaired Body Image (inverted score)**,** *MDI* multiple daily insulin injections, *CSII* Continuous subcutaneous insulin infusion

Moreover, CSII users had significantly more positive attitudes towards CSII according to the IPA total score (mean = 83; SD = 10.5) than MDI users (mean = 56.2; SD = 15.0) (*t* = 14.2; *p* < 0.001), as well as each subscale score (Fig. 1).

### Confirmatory factor analysis

**Fig. 2:**
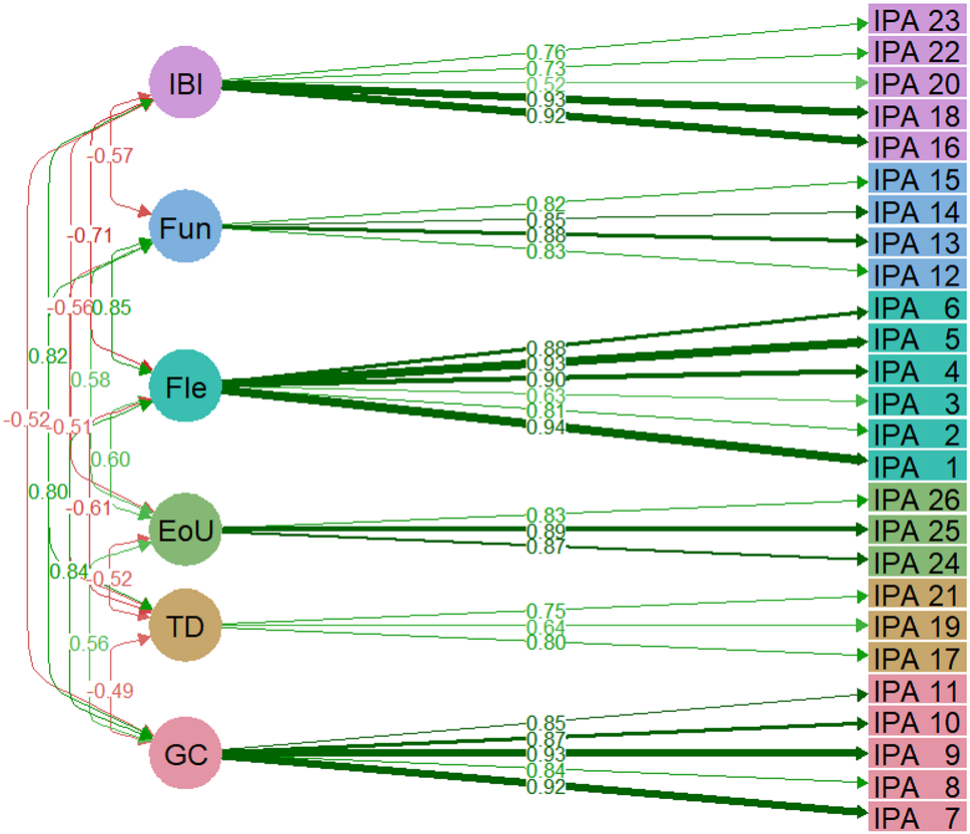
Structure of the Insulin Pump Attitudes (IPA) questionnaire resulting from the confirmatory factor analysis (standardized estimates). IBI = Impaired Body Image; Fun = Functionality; Fle = Flexibility; EoU = Ease of Use; TD = Technology Dependency; GC = Glycaemic Control. *GC* (IPA_7: With the insulin pump I can achieve better fasting glucose in the morning; IPA_8: With the insulin pump I can avoid hypoglycaemia more easily; IPA_9: With an insulin pump, I can avoid glucose/blood sugar fluctuations more easily; IPA _11: With an insulin pump I can achieve a better HbA1c; IPA _10: With the insulin pump I can better avoid large glucose/blood sugar excursions after a meal); *TD* (IPA _17: I'm constantly worried that my insulin pump might become defective; IPA _19: The pump alarms make me feel worried; IPA _21: I often worry about my insulin tubing becoming clogged or my insulin catheter kinking); *EoU* (IPA _24: The handling of my insulin pump is easy; IPA _25: The operation of the insulin pump was/is easy to learn; IPA _26: The handling of the insulin pump is intuitive); *Fle* (IPA _1: With an insulin pump, I have more Flexibility in my daily routine; IPA _2: With an insulin pump, I can do sports more spontaneously; IPA _3: With an insulin pump, I need less preparation for my sport; IPA _4: With the insulin pump, I can manage my glucose/blood glucose levels after a physical activity much better; IPA _5: With an insulin pump, I can respond to unexpected situations much better; IPA _6: With an insulin pump, I can organize my free time more flexibly); *Fun* (IPA _12: For the insulin pump, it’s important to me that I can program several basal rate profiles; IPA _13: For the insulin pump, it’s important to me that I can adjust the temporary basal rate as easily as possible; IPA _14: Regarding the insulin pump, it's important to me that it can be paired with a bolus calculator; IPA _15: For the insulin pump, it’s important to me that it can be paired with a continuous glucose measurement system); *IBI* (IPA _16: The insulin pump makes me feel more ill; IPA _18: It bothers me that I constantly have an insulin catheter in my body; IPA _20: Because of the insulin pump, others can immediately see that I have diabetes; IPA _22: The insulin pump disturbs my sleep; IPA _23: The insulin pump makes me feel less attractive)

Results of the confirmatory factor analysis indicate that the six-factor model proved to have a very good fit (CFI = 0.962, TLI = 0.956) with a reasonable error of approximation in the population (RMSEA = 0.087, 95% CI [0.078–0.095]; SRMR = 0.063). The structure of the IPA resulting from confirmatory factor analysis is shown in Fig. 2. Numbers above two-sided arrows denote correlations. Numbers above directed arrows indicate factor loadings, the higher the loadings the stronger the association of the item with the factor.

All correlations between factors were significant with *p* < 0.001: the strongest correlation was between Fle and Fun (*r* = 0.847) and between GC and Fle (*r* = 0.845), the lowest correlation was between GC and TD (*r* = -0.494).

Factor loadings (standardized estimates) were significant with *p* < 0.001 and they ranged from *β* = 0.52 of item 20 in the IBI factor (*“Because of the insulin pump, others can immediately see that I have diabetes”*) to *β* = 0.94 of item 1 in the Fle factor (“*With an insulin pump, I have more Flexibility in my daily routine*”). Factor loadings in GC, EoU, and Fun factor were higher than 0.8, while one item in each TD, Fle, and IBI factor had a factor loading below 0.65 (item 19 “*The pump alarms make me feel worried”*, item 3 “*With an insulin pump, I need less preparation for my sport”*, item 20 *“Because of the insulin pump, others can immediately see that I have diabetes”*, respectively).

### Internal consistency

Cronbach’s α of the total IPA scale indicated that the internal consistency was acceptable (α = 0.75; 95% CI [0.65–0.81]). For the subscales Flexibility and Glycaemic Control, the internal consistency was considered excellent (α = 0.92; 95% CI [0.88–0.94], and α = 0.91; 95% CI [0.86–0.93], respectively), for Functionality (α = 0.87; 95% CI [0.81–0.91]), Ease of Use (α = 0.86; 95% CI [0.81–0.90]), and Impaired Body Image (α = 0.84; 95% CI [0.81–0.88]) the consistency was good, while for Technology Dependency the consistency was questionable (α = 0.68; 95% CI [0.58–0.76]).

### Convergent and discriminant validity

The subscales Impaired Body Image and Technology Dependency measure negative aspects of the attitude towards the insulin pump, so their correlations were inverted: positive correlations indicate less Impaired Body Image and less Technology Dependency (Table 3).

**Table 3:** Correlations of the IPA subscales with the validation questionnaires

	GC	TD (inverse)	EoU	Fle	Fun	IBI (inverse)	IPA total score
DTSQ	0.271**	0.311**	0.360**	0.195**	0.166*	0.295**	0.309**
IT-DMSES F1		0.151*					
IT-DMSES F2	0.154*	0.210**		0.147*		0.193**	0.186*
PAID-5		-0.300**	-0.206**			-0.264**	-0.149*
PHQ-9		-0.304**	-0.159*			-0.247**	-0.154*
GAD-7		-0.232**				-0.210**	

*IPA* Insulin Pump Attitudes, IPA subscales *GC*  Glycaemic Control, *TD* (inv) Technology Dependency (inverted score), *EoU* Ease of Use, *Fle* Flexibility, *Fun* Functionality, *BI (inv)*  Impaired Body Image (inverted score)

*DTSQ* Diabetes Treatment Satisfaction Questionnaire, *IT-DMSES* The Italian version of the Diabetes Management Self-Efficacy Scale, *F1* diabetes management, *F2* lifestyle management, *PAID-5* Problem Areas in Diabetes Scale; *PHQ-9* Patient Health Questionnaire, *GAD-7* Generalized Anxiety Disorder

**Correlation is significant at the 0.01 level (two-tailed)

*Correlation is significant at the 0.05 level (two-tailed)

Diabetes treatment satisfaction was positively correlated with a more positive attitude towards the insulin pump therapy in general (rho = 0.31; *p* < 0.01), less Technology Dependency (rho = 0.31; *p* < 0.01), higher Ease of Use (rho = 0.36; *p* < 0.01), and less Impaired Body Image (rho = 0.30; *p* < 0.01).

Furthermore, less Technology Dependency was associated with lower diabetes distress (rho = -0.30; *p* < 0.01) and depression symptoms (rho = -0.30; *p* < 0.01).

## Discussion

Our study aimed to adapt the Insulin Pump Attitudes Questionnaire (IPA) from German to Italian, evaluate its psychometric properties, and explore correlations between attitudes towards CSII treatment and psychological aspects. Face and content validity were applied to translate and adapt the terms to improve understanding. A final IT-IPA questionnaire has been agreed upon by the research team. Confirmatory factor analysis has confirmed the following six factors of the original version: Flexibility, Glycaemic Control, Impaired Body Image, Functionality, Ease of Use, and Technology Dependency.

The IT-IPA resulted to be a valid and reliable questionnaire to be used with people with type 1 diabetes using or intended to use CSII. As expected and as reported by Bergis and colleagues [14], the IPA total score and the six-subscale scores were significantly different between CSII and MDI users, maybe due to the fact that people with diabetes on CSII therapy might perceive immediate benefits because they currently have more experience with technologies. This discriminant characteristic of the questionnaire might allow to identify potential new CSII users. According to Bergis and colleagues (2019) [14], the IPA questionnaire showed good internal consistency of all items and of items within each factor.

Our results also indicate that diabetes treatment satisfaction is positively correlated with a positive attitude towards insulin pump therapy in general. This point is particularly important because treatment satisfaction was associated with better Glycaemic Control in past studies [33, 34]. In addition, in our study, a positive attitude towards insulin pump therapy was associated with lower diabetes distress and depressive symptoms.

The observation that less Technology Dependency and Impaired Body Image and more Flexibility and Functionality were associated with lower diabetes distress and depressive symptoms is clinically meaningful given that psychological distress is associated with lower quality of life [35], higher occurrence of medical complications [36], family functioning [37], and lower adherence in people with diabetes [38].

On the other hand, some studies have previously reported that diabetes distress may be a barrier to the use of diabetes technology [39]. In particular, individuals perceiving more barriers to device use also report more diabetes distress and lower rates of device use [13, 40]. For instance, a recent survey explored barriers to device uptake in adults with T1D. In this study, those who reported more barriers to use devices had higher levels of diabetes distress and more negative attitudes about both technology in general and diabetes-specific technology [40]. Thus, modifiable barriers may be potential targets for intervention to increase uptake and prevent discontinuation or dropout.

We should acknowledge some limitations. First, the conclusions are limited by self-report assessment of clinical characteristics, which imply inherent inaccuracies in the reported estimates. Second, the cross-sectional design of our study limits the identification of longitudinal psychological factors impacting the attitudes towards insulin pump therapy and the ability to evaluate the questionnaire’s degree of reproducibility.

However, to our knowledge, this is the first scale in Italian assessing attitudes towards CSII therapy. In the next future, addressing psychological barriers and improving psychosocial outcomes in addition to clinical outcomes could be crucial to enhance patient acceptance of automated insulin delivery systems [40, 41]. Nonetheless, since the items of the questionnaire contain technical terminology, we suggest using the IPA with people having yet some knowledge of CSII therapy.

## Conclusions

The IT-IPA is a valid and reliable questionnaire evaluating attitudes towards insulin pump therapy, in particular experiences and expectations on using the pump. The questionnaire can be used for clinical practice and research to explore psychological barriers related to CSII dropout and to collect information on how best to support people with type 1 managing diabetes with an insulin pump. Further research is needed to compare attitudes towards insulin pumps and their relationship with glycaemic levels over time.
