# Correction to: Assessing attitudes towards insulin pump therapy in adults with type 1 diabetes: Italian validation of the Insulin Pump Attitudes Questionnaire (IT‑IPA questionnaire)

**DOI:** 10.1007/s00592-023-02090-3

**Published:** 2023-04-21

**Authors:** Rossella Messina, Liliana Indelicato, Marica Iommi, Maddalena Trombetta, Timm Roos, Norbert Hermanns, Annamaria Di Sipio, Maria Pia Fantini, Vincenzo Calvo

**Affiliations:** 1 Department of Biomedical and Neuromotor Sciences, University of Bologna, Via San Giacomo 12, 40126 Bologna, Italy; 2 University of Verona, Verona, Italy; 3 Dipartimento Di Medicina, Azienda Ospedaliera-Università Di Padova, Padua, Italy; 4 Research Institute of the Diabetes Academy Mergentheim (FIDAM), Bad Mergentheim, Germany; 5 Department of Philosophy, Sociology, Pedagogy, and Applied Psychology, University of Padova, Padua, Italy

**Correction to: Acta Diabetologica (2023) 60:687–695** 10.1007/s00592-023-02046-7

Author would like to update below corrections

The second author’s name is Liliana (name) Indelicato (surname) and not Indelicato Liliana as it appears at the moment.

Affiliation number 3 is incorrectly updated for all authors instead of updating only for the author Maddalena Trombetta.

The original article has been corrected.

